# Kynurenine Pathway Regulation at Its Critical Junctions with Fluctuation of Tryptophan

**DOI:** 10.3390/metabo13040500

**Published:** 2023-03-30

**Authors:** Ashley Newton, Luree McCann, Lu Huo, Aimin Liu

**Affiliations:** 1 Department of Chemistry, University of Texas at San Antonio, San Antonio, TX 78249, USA; ashley.newton@utsa.edu (A.N.); luree.mccann@my.utsa.edu (L.M.); 2 Department of Chemistry, Georgia State University, Atlanta, GA 30303, USA; lu.huo.1987@gmail.com

**Keywords:** tryptophan metabolism, enzyme complexation, protein structure, protein–protein interaction, enzymology, catabolic pathway, metabolic intermediates

## Abstract

The kynurenine pathway (KP) is the primary route for the catabolism of the essential amino acid tryptophan. The central KP metabolites are neurologically active molecules or biosynthetic precursors to critical molecules, such as NAD^+^. Within this pathway are three enzymes of interest, HAO, ACMSD, and AMSDH, whose substrates and/or products can spontaneously cyclize to form side products such as quinolinic acid (QA or QUIN) and picolinic acid. Due to their unstable nature for spontaneous autocyclization, it might be expected that the levels of these side products would be dependent on tryptophan intake; however, this is not the case in healthy individuals. On top of that, the regulatory mechanisms of the KP remain unknown, even after a deeper understanding of the structure and mechanism of the enzymes that handle these unstable KP metabolic intermediates. Thus, the question arises, how do these enzymes compete with the autocyclization of their substrates, especially amidst increased tryptophan levels? Here, we propose the formation of a transient enzyme complex as a regulatory mechanism for metabolite distribution between enzymatic and non-enzymatic routes during periods of increased metabolic intake. Amid high levels of tryptophan, HAO, ACMSD, and AMSDH may bind together, forming a tunnel to shuttle the metabolites through each enzyme, consequently regulating the autocyclization of their products. Though further research is required to establish the formation of transient complexation as a solution to the regulatory mysteries of the KP, our docking model studies support this new hypothesis.

## 1. Introduction

Tryptophan is an essential amino acid used for protein synthesis and is a precursor for generating metabolites with diverse biological activities throughout the body [1,2,3,4]. In humans, the bulk of L-tryptophan (L-Trp) is catabolized through the kynurenine pathway (KP). For years, this pathway has been known to be linked to depression and inflammation and is part of the depression theory known as the “kynurenine hypothesis” [5]. The KP metabolites are now recognized as etiological factors of depression after it was realized that the L-Trp level in the brain is not significantly altered, as stated in the initial proposal [6,7]. In T-cells, the KP enzymes and metabolites play an immunosuppressant role at checkpoints [8]. Many cancers hijack this regulation mechanism and overexpress the enzymes involved in the first step of the KP, indoleamine-2,3-dioxygenase (IDO) and tryptophan-2,3-dioxygenase (TDO), for immune escape [8,9,10,11]. Thus, IDO/TDO are among the most promising cancer immunotherapy targets pursued extensively in recent decades.

**Figure 1:**
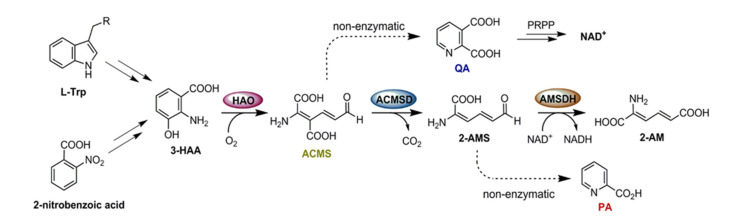
The L-tryptophan kynurenine pathway (KP) and the bacterial 2-nitrobenzoic acid (2-NBA) biodegradation pathway converge at the KP trio: HAO, ACMSD, and AMSDH.

Other than the first side product, kynurenic acid, a neuroprotective compound, the center of the catabolic route produces a series of neurologically active compounds such as 3-hydroxy-anthranilic acid (3-HAA), 3-hydroxykynurenine, kynurenic acid, and quinolinic acid (QA or QUIN) [12,13]. The center of the KP (Figure 1) branches out at several junctions due to the intrinsic instability of the metabolic intermediates 2-amino-3-carboxymuconate-6-semialdehyde (ACMS) and 2-aminomuconate semialdehyde (2-AMS) [14]. They spontaneously produce QA and picolinic acid (PA), respectively. To date, no allosteric regulation mechanisms are known for any of the KP trio systems, and based on measured mRNA profiling in a yeast system, the expression levels of the KP enzymes are not expected to vary based on L-Trp load [15]. In the absence of such mechanisms, the production of non-enzymatic products, QA and PA, should depend entirely on fluctuations in food intake. However, it has been shown in healthy individuals that the basal level of QA is maintained at low nanomolar concentrations despite L-Trp concentrations varying from sub-micromolar to hundreds of micromolar depending on the metabolic state [16,17,18,19,20,21,22]. In fact, QA becomes toxic to cells as its concentration exceeds this basal level, causing damage to spinal neurons at only 100 nM and to human central neurons at 350 nM [22]. This toxicity of QA can produce axon-sparing lesions analogous to those observed in Huntington’s disease [23,24]. Increased QA levels up to 20-fold are seen in HIV-infected patients’ cerebrospinal fluid (CSF), with even higher levels observed in those with HIV-1-associated encephalopathy [25]. Additionally, at heightened concentrations, QA can act as an endogenous agonist at *N*-methyl-D-aspartate (NMDA) receptors. Once activated, QA can cause abnormally long-lasting activity of the receptors, triggering an excessive influx of calcium ions into neurons, which can then provoke processes leading to neuronal damage, including the activation of proteases, formation of free radicals, and production of nitric oxide [22]. Considering the drastic impact of elevated QA levels on the body, further understanding of its regulation may prove useful for treating several neurological disorders. Since the concentrations of QA and PA are shown to be independent of metabolic state changes in healthy individuals but elevated in disease states, an intriguing question arises as to how the KP controls the production of non-enzymatically derived side products independent of metabolic state changes [26,27].

Although this pathway has been well-established for its biological significance, regulatory mechanisms of the KP have remained elusive. Several bacterial systems possess the gene clusters encoding the KP proteins for aerobic tryptophan degradation [28,29]. This enzymatic junction solicits interest, considering that in bacteria, the 2-nitrobenzoic acid (2-NBA) degradation pathway converges with the KP at this trio [30]. The structural and mechanistic understanding of the individual enzymes first came from the bacterial enzymes and later extended to their human counterparts. This paper discusses the molecular mechanism for regulating the KP, highlighting the competition between enzyme-mediated reactions and the non-enzymatic autocyclization of their substrates and products by focusing on the activity of a unique subset of the KP enzymes (Figure 1), hereafter referred to as the KP trio. These are (i) a non-heme iron-dependent dioxygenase HAO (E_1_), (ii) a Zn-dependent decarboxylase ACMSD (E_2_), and (iii) an NAD^+^-dependent semialdehyde dehydrogenase AMSDH (E_3_). In what follows, the latest comprehension of the structure and mechanism of each trio enzyme will be summarized, followed by a new hypothesis on how the non-enzymatic products might be maintained at high levels of KP metabolites in a metabolic-independent manner.

## 2. HAO: The Phenyl Ring-Breaking Oxygenase in the KP Trio

The first enzyme of the KP trio, HAO, is a non-heme iron-dependent enzyme that produces an unstable metabolite ACMS [31], whose chemical structure has only recently been structurally determined to be in the 3*E*,5*Z*,2*t*,4*t* conformational state among the 32 possible enol and keto tautomers [32]. ACMS can either spontaneously cyclize to form QA or be decarboxylated to form 2-AMS via an ACMSD-mediated reaction [33,34]. This reaction is eye-catching as QA is one of the only known endogenous metabolites, the other being kynurenic acid, capable of explicitly modulating the activity of NMDA receptors when its concentration is above the basal level [1,17,18,19,20,21,22,23,35,36]. Elevated levels of QA in the CSF are seen in patients with neuropsychiatric and neurodegenerative diseases, including anxiety, depression, Alzheimer’s, and Huntington’s diseases [23,24,37,38,39,40,41,42,43,44]. Additionally, QA is the universal precursor for NAD^+^ biosynthesis, and further understanding of the formation of QA may provide much-needed insight into the regulation of NAD^+^ *de novo* synthesis [28,32,45]. As NAD^+^ is known to enhance mitochondrial function and improve health, this proves to be an attractive avenue of research within the KP [4].

**Figure 2:**
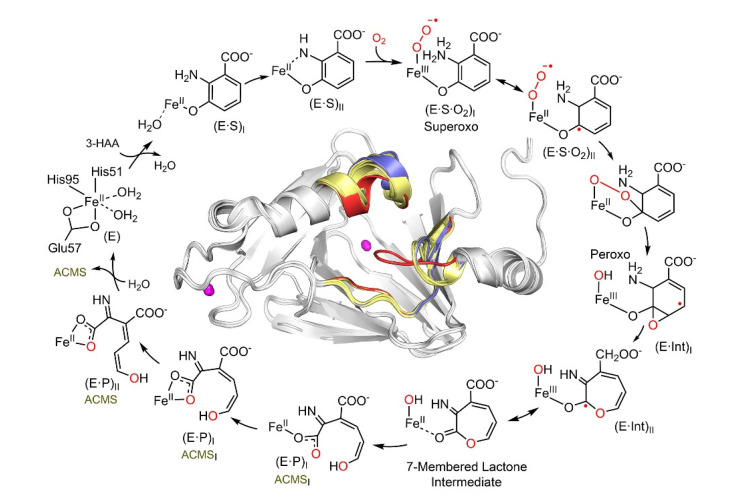
The HAO catalytic reaction cycle is characterized by *in crystallo* chemical reaction. The structurally characterized intermediates include monodentate E•S complex, bidentate E•S complex, Fe-bound superoxo, alkylperoxo, ɛ–lactone, monodentate, and the reaction product bidentate 3*E*,5*Z*,2*t*,4*c*–enol tautomer of ACMS, as well as bidentate 3*E*,5*Z*,2*t*,4*t*–enol tautomer of ACMS. The (E•Int) radical intermediates are implicated by the single-crystal EPR spectroscopy, although not structurally resolved. The inset shows the overall structure and dynamic loop change near the catalytic site which occurs during catalysis. The loops of substrate-free, bidentate E•S complex, and subsequent intermediates are colored in blue, red, and yellow, respectively. For more details regarding the catalytic pathway intermediate structures and loop dynamics, see References [32,47].

Aside from being a promising drug target, HAO participates in exciting chemistry. This enzyme cleaves the aromatic ring of 3-hydroxyanthranilate (3-HAA) adjacent to the substitution groups and activates and inserts dioxygen between C3 and C4 by a non-heme iron center [46]. Despite relatively low local O_2_ concentrations and diffusion rates, HAO can rapidly bind O_2_ by undergoing a substantial conformational change and possessing significant loop dynamics during catalysis [47]. By doing so, the enzyme can quickly and efficiently bind the hydrophilic substrate 3-HAA, and then, the hydrophobic substrate O_2_. This novel feature of loop dynamics is not limited to only class III estradiol dioxygenases such as HAO. It may have broad implications for understanding how enzymes work at a high turnover rate while accommodating two substrates for rapid binding with disparate polarities [47]. A deeper understanding of the mechanism of enzyme action has been achieved through time-resolved reactions performed *in crystallo* and monitored by single-crystal spectroscopies and X-ray diffractions to trap and characterize intermediates unseen in the solution-state reaction [32]. A total of seven crystal structures of sequentially observed reaction intermediates provided step-by-step information on how 3-HAA interacts with the catalytic Fe center during the initial stages of catalysis, leading to a fuller view of the HAO catalytic cycle (Figure 2). The catalytic pathway begins with monodentate and then bidentate 3-HAA coordination, binding, and activation of dioxygen, O–O bond cleavage, oxygen insertion, Criegee rearrangement causing ring expansion, a second oxygen transfer with ring opening, and finally, an enzyme-assisted change of the product conformation [32]. Prior to this work, the exact conformation of ACMS produced by HAO remained unknown for decades due to its short lifetime and 32 possible conformers. However, the crystal structures of the catalytic cycle intermediates reveal that HAO possesses in situ isomerase activity immediately after the dioxygenase function, so it may be able to maintain ACMS in a more stable conformation. As such, HAO counteracts autocyclization, preserving a portion of the metabolic flux for other downstream enzymes in the pathway.

Three loop regions surround the catalytic iron center, which forms “open” and “closed” conformations at the HAO active site to carry out its dynamic chemistry (Figure 2 inset). These active site loops move upon substrate binding and are implicated as potential interaction surfaces with ACMSD. These loops facilitate the binding of a considerably hydrophilic substrate and enable dynamic shifting to create a more hydrophobic environment to capture molecular oxygen [32]. Substrate and product exchange when HAO is in its “open” state, and oxygen is incorporated in the “closed” state [47]. The substrate analog 4-Cl-3-HAA has been shown to be an effective suicide inhibitor of HAO [48]. Its binding locks HAO in the closed conformation [47]. The locked HAO conformation is hypothesized to facilitate protein–protein interactions with ACMSD, showing a dose-dependent decrease in extracellular QA concentration over time [49].

## 3. ACMSD: A Novel Metal-Dependent Non-Oxidative Decarboxylase

ACMSD is a zinc-dependent decarboxylase at the heart of the KP [34,50,51]. ACMSD is most expressed in kidney and liver cells, and its inhibition enhances mitochondrial function and increases cellular levels of NAD^+^ and NADH [33]. It performs a metal-dependent, non-oxidative decarboxylation of its substrate ACMS [52,53], proceeding through a metal-bound hydroxide with the assistance of non-covalent interactions with arginine residues in the active site. ACMSD competes with the non-enzymatic cyclization of ACMS into QA by removing the β-carboxylic group of ACMS to form 2-AMS, which is converted to glutaryl-CoA and acetate through a series of glucogenic and ketogenic reactions before continuing to the citric acid cycle for oxidation and energy production. ACMSD holds a key position at the juncture of the autocyclization and KP and controls the fluctuation of the metabolites in both pathways. A significant trait of the ACMSD reaction is that the substrate and its product are unstable, which has made studying its reaction mechanism complex [31]. Therefore, QA levels are directly affected and regulated by ACMSD and could be elevated by inhibition of ACMSD [4,54,55]. QA is an essential precursor to the universal biological oxidant NAD^+^ and the reductant/energy carrier NADH. However, QA is an agonist of the NMDA receptors, and elevated cellular levels of QA are related to neuronal excitotoxicity and apoptosis. Therefore, its production is highly regulated in cells [44,56,57,58]. Further study on the regulation and inhibition of ACMSD and its role in QA levels and inflammatory response could elucidate the cause of many neurodegenerative diseases [13].

**Figure 3:**
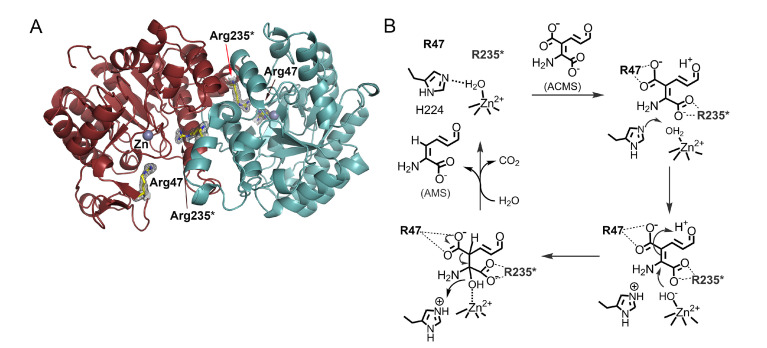
(**A**) The crystal structure of human ACMSD shows a homodimer with an essential Arg235 in the active site from a neighboring subunit (PDB: 4OFC). (**B**) The catalytic cycle of ACMS decarboxylation. For more information, see References [59,60,61,62].

As stated previously, ACMSD is a zinc-containing protein (Figure 3A). It is catalytically active in other divalent metal ions, such as cobalt(II) or iron(II), albeit at different efficiencies [34]. An active-site histidine functions as an acid-base catalyst to help generate a nucleophilic zinc-bound hydroxyl group that attacks the ACMS substrate, opens its C2 and C3 double bond, generates a C3-centered tetrahedral intermediate, and destabilizes the carboxyl group associated with C3 (Figure 3B) [59,60]. ACMSD can exist in solution as a tetramer, dimer, and monomer before degradation, and its activity is oligomeric-state dependent [60,61]. The monomer is catalytically inactive due to the requirement for neighboring arginine residues in its active site [60,61,62]. Even though ACMS is a metal chelator, mechanistic studies suggest that it does not directly ligate to the zinc ion but binds to the active site through non-covalent interactions with two arginine residues [60,62]. Each monomer only contains half of the necessary active site arginine residues, which would explain why the monomer is inactive [60]. When the enzyme is in its dimeric form, the neighboring arginine residues can work together to bind the substrate [62]. Mutation of either arginine residue from the neighboring protomer in the monomer–monomer interface eliminates the activity of the enzyme, which could explain the requirement of the dimeric and higher-order oligomers for catalytic activity and regulation of its unstable substrate and product [61]. ACMSD can self-assemble into its homodimer, tetramer, and higher-order structures. The dimer is the active unit, but the tetramer and higher-order structures show more specific activity [61]. This dynamic oligomerization, which potentially regulates ACMSD activity, is affected by pH, ionic strength, and other electrostatic interactions [13,61]. The homodimer of ACMSD utilizes “half-of-sites” reactivity, meaning that one of its active sites is well ordered and performs more efficiently than the other. The tetramer and higher-ordered structures may function to stabilize both active sites of the dimer. ACMSD’s ability to self-assemble into multi-oligomeric states provides insight into how it can efficiently regulate the branching of two metabolic pathways while maintaining an unstable substrate [61]. Recently, a potent inhibitor of the enzyme, 3-[[[5-cyano-1,6-dihydro-6-oxo-4-(2-thienyl)-2-pyrimidinyl]thio]methyl]phenylacetic acid (also known as TES-1025), has been identified, which provides a powerful utility for evaluation of the therapeutic potential of ACMSD inhibition in treating disorders with perturbed NAD^+^ homeostasis or supply [4,54,63]. The phosphorylated glycolytic intermediates 1,3-dihydroxyacetonephosphate (DHAP) has been found to be an inhibitor of ACMSD and a structural study reveals it binds to human ACMSD, and hence, a regulatory link between *de novo* NAD^+^ biosynthesis and glycolysis is suggested [64]. However, the structure is in a catalytically incompetent monomeric form. In the absence of biochemical data, it remains unestablished for the proposed link to glycolysis.

## 4. AMSDH: An NAD^+^-Dependent Dehydrogenase in the KP Trio

The final enzyme of the KP trio is the previously elusive human AMSDH, an NAD^+^-dependent semialdehyde dehydrogenase that catalyzes the oxidation of 2-AMS to 2-AM. AMSDH is the first energy-harvesting step of the KP, which facilitates the NAD^+^-dependent oxidation of 2-AMS to 2-AM and competes with the spontaneous non-enzymatic cyclization of 2-AMS to PA, whose overproduction is toxic [65]. Until 2018, the last gene and corresponding enzyme identified in the KP was ACMSD, leaving ten missing steps after product 2-AMS. Previously, the gene encoding human protein was ALDH8A1, which was misassigned to that of retinal dehydrogenase in a previous study based on its ability to oxidize 9-*cis*-retinal faster than all-*trans*-retinal. Thanks to a thorough characterization of the bacterial version of AMSDH [66,67], the human protein ALDH8A1 was reexamined. A recent study utilizing a coupled enzyme assay verified by 2D-NMR and a stable alternative substrate, 2-hydroxymuconic 6-semialdehyde (2-HMS), demonstrates ALDH8A1’s ability to catalyze the dehydrogenation of semialdehyde substrates better than *cis*-retinal. These findings are compared to the oxidation product of the natural substrate 2-AMS, and resonances and cross-peaks establish ALDH8A1 as human AMSDH [14].

**Figure 4:**
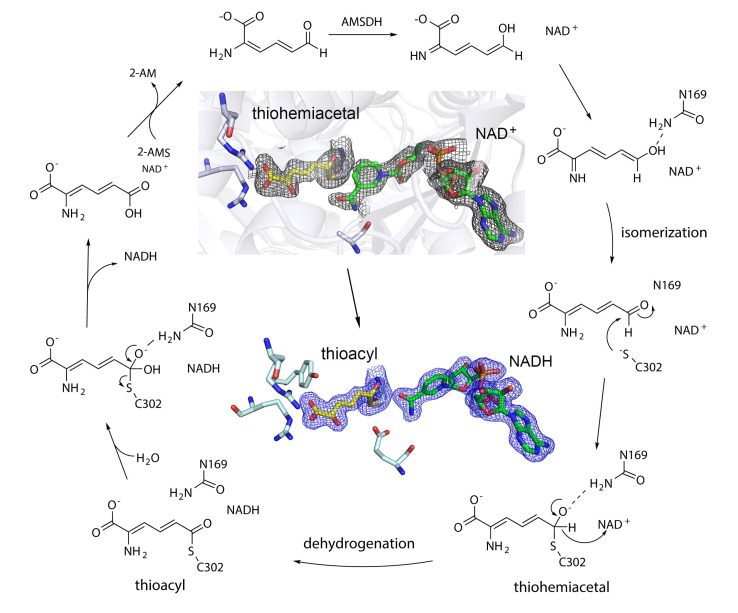
Catalytic mechanism of AMSDH based on a series of structurally captured intermediates. Like HAO, AMSDH has an innate isomerase activity. The inset shows the crystal structures of highly reactive key intermediate thiohemiacetal observed from E268A mutant and thioacyl observed in both wild-type enzyme and E268A mutant. For more information, see References [66,67].

Crystallographic studies of AMSDH from its bacterial analog explicate some of the elusive chemistry of aldehyde dehydrogenases. The first set of crystal structures for AMSDH included the resting state, one binary (bound with co-substrate NAD^+^), two ternary complexes (bound with NAD^+^ and the highly unstable primary substrate 2-AMS or an alternate substrate 2-HMS), a covalent thioacyl intermediate, and a tetrahedral thiohemiacetal intermediate [66]. These early crystal structures indicated an *E*/*Z* isomerization of the substrate in the enzyme active site before an *sp*^3^-to-*sp*^2^ transition during enzyme-mediated oxidation (Figure 4) [66]. The significance of a conserved asparagine, Asn-169, has been probed by generating Ala, Ser, Asp, and Gln mutants. The results have been substantiated theoretically and experimentally with a “pitcher-and-catcher” mechanism driving the isomerization before dehydrogenation [67]. Similar to ACMSD, the active site of AMSDH contains two arginine residues that help to stabilize the carboxyl group of its substrate. The active site also includes a cysteine residue that acts as a catalytic nucleophile, a glutamate that acts as a general base, and a conserved asparagine residue that is hypothesized as the oxyanion hole by stabilizing the transition-state negative charge on the C6 oxygen of the substrate through hydrogen bonding. This asparagine interaction appears to be essential in the rate-limiting steps of the reaction but is not heavily involved in catalysis. Mutation of this asparagine residue results in loss of dehydrogenase activity. The two arginine residues function as the pitcher in the pitcher-and-catcher mechanism, driving the isomerization activity utilizing electrostatic interactions with the substrate. The asparagine residue functions as the catcher in the mechanism, stabilizing the substrate in its necessary tautomeric conformation for dehydrogenation. This isomerization decreases steric hindrance and the distance between the C6 of the substrate and the sulfur of the cysteine residue for the subsequent step of the reaction. The utilization of hydrogen bonding to stabilize the highly unstable substrate and intermediates, coupled with an isomerization reaction to increase the efficiency of catalysis, prevents the spontaneous autocyclization of the substrate inside the enzyme and allows AMSDH to compete with the non-enzymatic production of PA [67].

## 5. Transient Enzyme Complex as a Possible Pathway Regulatory Mechanism

In a healthy individual, the QA level remains at a steady basal level in the nanomolecular range [68,69]. It is independent of the metabolic cycle of absorptive (fed), postabsorptive (fasting), and starvation. It has been reported that the ratio between ACMS-to-QA and ACMS-to-2-AMS is 1:72 in healthy adults [27]. However, the paradox is that QA and PA are formed non-enzymatically and are expected to depend on metabolic states. At the low level of L-Trp in cells, ACMSD and AMSDH can effectively compete with the spontaneous reactions of ACMS and 2-AMS when they are present at low levels. The substrate concentrations near the *K*_M_ value of the enzymes can effectively tune the catalytic rate. In contrast, these kynurenine metabolites should unavoidably produce more QA and PA at high levels.

The mechanistic enzymology of each enzyme of the KP trio has been extensively studied. Still, a knowledge gap surrounding competition between enzymes and the non-enzymatic autocyclization of their substrates and products. In a highly active metabolic state, the three enzymes must be able to steer the metabolic flux towards the enzyme-controlled route, preventing excessive accumulation of the side products. Adding to the complexity, they must also allow a steady, low-level production of QA for *de novo* biosynthesis of NAD^+^ in humans [1]. Few explanations emerge. One possibility is that these three enzymes are expressed in large quantities shortly after a meal and then broken down in the fasting state. However, this would be highly resource-demanding, which is not supported by the mRNA profiling study [15]. In fact, no data thus far show a significant metabolic-state-dependent fluctuation of the KP enzyme levels. Another possible regulatory mechanism is tuning the catalytic activity of the center enzyme of the KP trio, ACMSD. As discussed above, its enzymatic activity depends on its quaternary structure, with which it can be modulated through its oligomeric states from inactive (monomer) to active (dimer) and even more active (tetramer and high-order structures) forms [61]. However, no evidence shows a substrate-tuned equilibrium shift among these distinct forms. Thus, such an ACMSD quaternary structure-dependent KP regulation is limited, perhaps only to adapting to environmental changes such as pH variations. No allosteric-type-pathway product feedback inhibition mechanisms are known for the KP trio, either. Hence, the metabolic-state-independent nature of the QA and PA levels remains perplexing and cannot be rationalized with knowledge of each individual enzyme.

**Figure 5:**
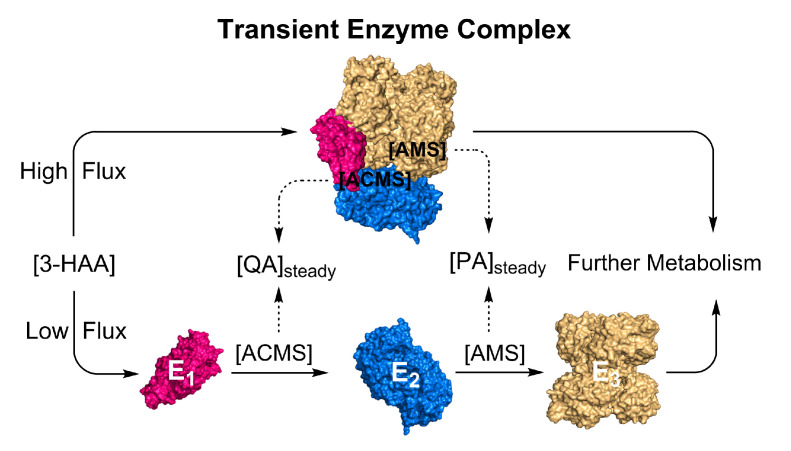
Illustrated hypothesis: formation of substrate-induced transient enzyme complex at high metabolic flux for regulating non-enzymatic reactions. E_1_: HAO, E_2_: ACMSD, and E_3_: AMSDH.

To resolve the puzzle, we propose the metabolite-induced formation of a transient enzyme complex as a mechanism to regulate the metabolite partitioning between enzymatic and non-enzymatic routes in the KP (Figure 5). In the scenario where the kynurenine metabolites exceed a certain level, they will trigger the KP trio to change their conformations and interact with each other, forming enzyme–enzyme complexes, either pairwise E_1_-E_2_ and E_2_-E_3_ complexes or an HAO-ACMSD-AMSDH (E_1_-E_2_-E_3_) ternary complex, to shuttle the unstable metabolites from one active site to another, deliberately reducing the spontaneous reaction rates during high levels of L-Trp metabolites. In the free solution, QA and PA production depends on the L-Trp metabolite levels, temperature, and pH. Inside a protein tunnel, ACMS and 2-AMS will not only be in an increased hydrophobic environment but also one with restricted conformational changes to reduce their tautomerizations necessary for achieving autocyclization. At the fed state, the only plausible mechanism for avoiding QA and PA overproduction and maintaining them at a low basal level is guiding the metabolic precursors from one enzyme to the next, thereby sequestering them from the bulk solvent. At high levels of the KP metabolites, it is likely that this enzyme trio is functionally intertwined and that their close connectivity arises from their ability to regulate each other’s activity and product distribution through direct protein–protein interactions.

**Figure 6:**
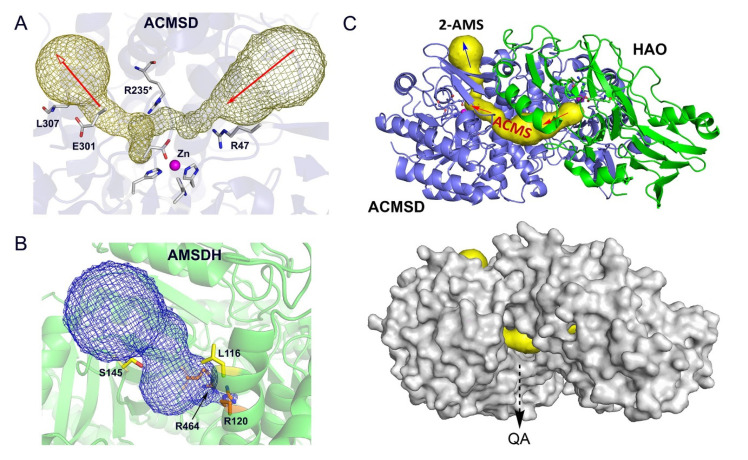
*In silico* docking model of HAO-ACMSD. (**A**) ACMSD has two tunnels that intersect at the metal center. (**B**) *Pf*AMSDH substrate binding channel (PDB: 4I25). (**C**) An HAO (green) and ACMSD (blue) docking model built from their corresponding crystal structures. The substrate tunnel is highlighted in yellow. A space-filling of the docking model reveals a leak point that allows spontaneously formed QA to exit.

The center enzyme of the trio, ACMSD, presents two potential tunnels in its structure (Figure 6A). AMSDH also has a cavity for substrate binding that is validated by the ternary-complex crystal structure of the bacterial enzyme (Figure 6B). To investigate this connectivity further, a docking model was built from corresponding human ACMSD and HAO crystal structures (PDB entries 4IGN and 2QNK, respectively [70,71]) by using the ZDOCK utility and server (http://zdock.umassmed.edu, accessed on 22 February 2023) [72]. This *in silico* docking model, shown in Figure 6C, reveals a tunnel through which 3-HAA can enter and be guided to ACMSD to be converted to ACMS, which could then be directed to AMSDH to form 2-AMS. Importantly, this model highlights a leak point for ACMS to spontaneously form QA, which is essential for the *de novo* biosynthesis of NAD^+^. This inner channel allows the enzymes to out-compete the autocyclization of their substrates while still tolerating a necessary amount of autocyclization to maintain proper levels of QA and PA. Thus, the results of *in silico* study support the hypothesized pathway-regulation model delineated in Figure 5.

## 6. Concluding Remarks

Transient physical association between enzymes has become increasingly appreciated as a cardinal feature of metabolic systems [73]. We envision such a transient enzyme complexation occurring and being of benefit in the following two scenarios. First, a transient enzyme complex may facilitate enzymatic biotransformation, which is energetically unfavorable and does not occur in a test tube. The reaction becomes an intermediary step in an overall reaction when coupled with a preceding enzyme. The energy released from the prior reaction drives the conformational change of the target enzyme and delivers power to the reaction that otherwise would not occur. Second, the transient, substrate-induced enzyme–enzyme associate could serve as an effective regulatory mechanism for a metabolic pathway involving unstable metabolites, which is a common scenario. In this latter case, the enzymes compete with non-enzymatic chemical reactions, and the complexation is expected to slow down the spontaneous reaction rates. However, the mechanisms by which enzymes of a metabolic pathway interact with each other and the contribution of such interactions to the internal regulation of various metabolic pathways remain unresolved. Investigating transient enzyme assemblies presents a unique scientific challenge, as such enzyme complexes do not typically exhibit sufficient stability to be isolated and characterized. This scenario is especially pertinent in the case of the L-Trp degradation pathway in the KP trio segment, wherein each metabolite is unstable. Once established, such a mechanism will expand the current repertoire of metabolic pathway regulatory strategies and bring the field of mechanistic enzymology one step closer to understanding enzymes in their native, complex cellular environment.

## Data Availability

All data relevant to this article are included in this publication.
